# Self-reported visual impairment and depression of middle-aged and older adults: The chain-mediating effects of internet use and social participation

**DOI:** 10.3389/fpubh.2022.957586

**Published:** 2022-11-17

**Authors:** Wenbo He, Peiyi Li, Yinyan Gao, Jiuhong You, Jiangeng Chang, Xing Qu, Wei Zhang

**Affiliations:** ^1^Institute of Hospital Management, West China Hospital of Sichuan University, Chengdu, China; ^2^Saw Swee Hock School of Public Health, National University of Singapore, Singapore, Singapore; ^3^Department of Anesthesiology, West China Hospital, Sichuan University, Chengdu, China; ^4^Laboratory of Anesthesia and Critical Care Medicine, National-Local Joint Engineering Research Center of Translational Medicine of Anesthesiology, West China Hospital, Sichuan University, Chengdu, China; ^5^The Research Units of West China (2018RU012)-Chinese Academy of Medical Sciences, West China Hospital, Sichuan University, Chengdu, China; ^6^Xiangya School of Public Health, Central South University, Changsha, China; ^7^School of Rehabilitation Sciences, West China Hospital of Sichuan University, Chengdu, China; ^8^West China Biomedical Big Data Center, West China Hospital of Sichuan University, Chengdu, China

**Keywords:** vision impairment, depression, internet use, social participation, chain-mediating effects

## Abstract

**Background:**

Visual impairment (VI) is a strong predictor of depression in middle-aged and older adults. However, the underlying mechanisms and pathways have not been well characterized. The purpose of this study was to determine whether Internet use and social participation mediate the effects of self-reported VI on depression.

**Methods:**

The study used the fourth wave of cross-sectional data from the China Health and Retirement Longitudinal Study, including 19,766 Chinese adults. Depression was assessed according to the CES-D 10 International Scale. Logistic regression models were used to examine the relationship between self-reported VI and depression. While adjusting for relevant covariates, the PROCESS macro (model nos. 6 and 91) was used to assess the chain-mediating effects of Internet use and social participation.

**Results:**

A total of 17,433 respondents were included in this study. The CES-D 10 results showed that 7,327 middle-aged and older adults had depressive symptoms, of whom 39.5% were male and 10.2% were ≥75 years old. 32.1% of respondents self-reported VI. Regression analysis showed a positive association between VI and depression, while Internet use and social participation had a negative predictive effect on depression. In the mediation analysis, the social participation pathway contributed the most to the total effect, accounting for 52.69% of it. The proportion of Internet use is 37.72%. When these two mediators were considered together in the full model, they accounted for 9.58% of the total effect of VI on depression.

**Conclusion:**

Internet use and social participation were important mediators that mitigated the effects of VI on depression. Combined with previous evidence, online activities such as e-health and m-health can effectively promote disease monitoring and diagnosis, and various offline social participation activities can also play a role in regulating emotions. Therefore, Internet use and social participation factors may serve as relevant entry points for the development of intervention programs that may further improve the mental health of the visually impaired.

## Introduction

The term visual impairment (VI) refers to self-reported blindness or difficulty with distance/near vision. According to the definition proposed by the World Health Organization, which used the International Classification of Diseases 11 (ICD-11) definition of VI and blindness, a person is said to be visually impaired if he or she presents with worse than 3/60 of the visual acuity (VA) of the better eye. In this revised definition, near VI is also included; it is defined as presenting with poorer near VA than N6 with the available correction (1). As recently estimated by the Global Burden of Disease Study, it is suggested that VI ranked second among all contributing causes of years of disability worldwide, ahead of depression (2). VI affects more than 250 million people worldwide, 90% of them live in low- and middle-income countries, and 82% are aged 50 years or older (3). As the most populous country in the world, China has a large number of people with VI or blindness which will increase substantially (4). The prevalence of vision loss increased with age, and 61% to 67% of adults aged over 35 years are affected by VI in China (5). The impacts of VI and blindness are wide reaching, such as an increased risk of falls (6), cognitive impairment and dementia (7), depression (8), and loss of independence (9). Clinically significant sub-threshold depression has been found in one-third of older adults with VI, approximately two times as high as the lifetime prevalence rates in older adults without VI, where depressive symptoms are present roughly in 15% (10, 11). Depression is a serious medical condition, and even mild symptoms may affect the quality of life (12). Therefore, the treatment of depression has increasingly gained attention within eye care settings as shown by numerous mental healthcare programs that have been tested and often found effective (13).

Behavioral factors are the leading cause of ill health worldwide. More health knowledge and self-efficacy positive health behavior change may promote better depression prevention and management (14, 15). According to the behavior change theory, effective Internet intervention could produce changes in health behaviors *via* effective Internet use and adherence (16, 17). The Internet is a powerful tool that can connect the older population with online resources, online social activities, and other people (18). Substantial efforts have been made by researchers across the world to utilize the power of the Internet to promote health outcomes. Older cohorts could use the Internet to reconcile their feeling of loneliness by engaging themselves in online activities and interactions with others and strengthening their connectedness with social members. For instance, chatting and watching the news could increase their sense of independence, while entertainment-oriented use of the Internet such as playing games could provide them with emotional support and relieve the pressure of life (19). Such enhanced connection through the Internet would alleviate loneliness and enhance the sense of social inclusion for older adults (20). Similarly, Internet use frequency has also been evidenced with a significant potential to alleviate older adults' depression (21). Social media and Internet use frequency had been detected a significant relationship with Chinese netizens' adoption of Web-based healthcare advice and changes to their preventive behaviors. Thus, frequency may be having a more weighted effect on health behaviors change (22). Additionally, the previous study has provided support for the beneficial role of social participation, such as joining community activities in preserving mental health (23, 24). Potential advantages to engagement in community activities and higher levels of social cohesion were associated with better mental health status among the older population (25, 26). In addition, studies have shown that social media is an effective tool for mobilizing people to participate in social movements, which further promotes people's willingness to engage in social activities (27). The use of the Internet is defined by its online interactivity, which allows users to also contribute content and receive feedback on it from others through comments or “likes.” This promotes users' willingness to engage in social action by increasing their sense of psychological empowerment (28).

Vision disorders with activity limitations were always associated with decreased social participation (29). Initially, visually impaired people experienced significant barriers to using mobile phones for other purposes than making calls; however, with the introduction of the iPhone in 2009 and improvements in services, such as voice capabilities, people with VI were able to expand the use of their phones beyond just making phone calls (30). In addition, the use of the Internet has been suggested to be a means of providing interaction Opportunities and reducing negative emotions for the VI group, the relationships between Internet use, social participation, and depression in older adults with vision disorders, which might be more complex than previously reported for normal vision population. Therefore, it is necessary to investigate the impact of Internet use and social participation on negative emotions to inform policies and programs to help seniors with vision disorders optimize their quality of life while aging. However, studies related to the impact of Internet use and social participation on mental health for people with VI have not been actively conducted, and also, the impact intensity and interaction have not yet been investigated. Given the Chinese rapidly growing aging population, we aim to investigate the relationship between VI and depression through a nationwide survey and further clarify the pathway of mediating effects of Internet use and social engagement in it.

## Methods

### Data and sample

The data collected for this study were from wave 4 of the China Health and Retirement Longitudinal Study (CHARLS). In brief, the CHARLS is a longitudinal study assessing the health, social, and economic status of a nationally representative sample covering 450 villages and 150 counties in 28 provinces in China (31). The CHARLS collected high-quality data through one-on-one interviews with a structured questionnaire, using multilevel stratification probabilities proportional to sample size to select residents aged 45 years and older from constituting a nationally representative sample. The fourth wave of data was collected in 2018 using an on-site survey, yielding a total sample of 19,766 adult respondents aged 40–98 years (32). The variables related to Internet use included in this study were newly added during wave 4 data collection, so only wave 4 data were used in this study for analysis.

### Inclusion and exclusion criteria

In the Chinese population, uncorrected refractive error was relatively less affected by population aging, and the populations affected are becoming younger (4). Therefore, we expand the scope of the analysis by including people ≥45 years and sub-analysis differences between middle-aged and older groups. In this study, 45–64 years were defined as the middle-aged group and ≥65 years were defined as the older group.

We excluded participants who were aged < 45 years; those with or who had intellectual disabilities, malignancies, and memory-related disorders, as this group may have experienced recall bias due to poor physical or mental status during data collection, thus becoming a confounding factor in the study results (32); and those who did not complete the depression test in wave 4. Finally, a total of 17,433 eligible individuals remained in the study.

## Measures

### Vision impairment

In the CHARLS, respondents were asked about vision (“Are you blind or unable to see at all?”, “Are you usually wear glasses or corrective lenses?”, “Seeing things at a distance,” “Seeing things up close,” “Have you ever had ever been treated for glaucoma or cataract?”). In this study, if middle-aged and older adults reported one of “blindness, poor-looking far away, poor-looking near, cataracts, and glaucoma,” they were judged to have VI (5). In order to ensure the accuracy of the characteristics of the included population, we excluded the item “Are you usually wear glasses or corrective lenses?” in the screening of the population with VI by referring to the ICD-11 definition of VI.

### Internet use

Respondents were asked whether they had used the Internet in the last month (Yes = 1 and No = 0) and frequency of Internet use (Never = 0, Not regularly = 1, Almost every week = 2, Almost daily = 3). At the same time, we judge the specific situation of their Internet use through the analysis of several common Internet behaviors, including online chatting, mobile payments, reading news, watching videos, playing games, financial management, and others. Each type of network function usage is assigned a value of 1, and Internet use as a continuous variable was assigned a score range of 0–7.

### Depression

The score of depression was measured with 10 questions from the Center for Epidemiologic Studies Depression Scale (CES-D10)(33, 34). The CES-D10 was derived from the original version of the 20-item CES-D, and it was highly validated for use in general populations and indicated adequate reliability and validity for the middle and older population in China (35). Respondents were asked how frequently in the last week they: were bothered by things; had trouble keeping on things; felt depressed; felt everything was an effort; felt hopeful about the future; felt fearful; had restless sleeping; were happy; felt lonely; and could not get going. Responses ranged from 0 to 3, where 0 = < 1 day, 1 = 1–2 days, 2 = 3–4 days, and 3 = 5–7 days, and were summed to create a total score ranging from 0 to 30. A binary measure for scoring 10 or greater was considered to have depressive symptoms (36, 37).

### Social participation

In the CHARLS, respondents were asked to choose which social activities they participated in during the past month, including (1) interacting with friends, (2) playing Ma-Jong, playing chess, playing cards, or going to the community club, (3) providing help to family, friends, or neighbors who do not live with you and did not pay you for the help, (4) going to a sport, social, or other kinds of the club, (5) taking part in a community-related organization, (6) doing voluntary or charity work, (7) caring for a sick or disabled adult who does not live with you and who did not pay you for the help, and (8) attending an educational or training course. We generated social participation as a binary variable (participating in at least one of these activities in the past month = “1” vs. no participation = “0”). Those who reported any of these activities were then asked a follow-up question about how often in the last month did they do (Almost daily = 3, Almost every week = 2, Not regularly = 1). We multiply the number and frequency of social participation to generate a multi-categorical variable for the social participation intensity (0 = No, 1–2 = Mild, 3–4 = Moderate, Above 4 = Heavy). Previous research has verified the reliability of this evaluation index (38).

### Covariates

A parsimonious set of sociodemographic factors were included: (1) gender, (2) age (45–54, 55–64, 65–74, and ≥75 years), (3) area (central city/town, urban–rural integration zone, and rural), (4) education level (below the middle school, below college degree, and college degree and above), (5) marital status (married vs. not married including unmarried, divorced, and widowed), (6) health status (self-rated health that was obtained by asking respondents, “Would you say your health is very good, good, fair, poor, or very poor?” We redefined “very good,” “good,” and “fair” as good health and assigned a value of 1; we redefined “poor” and “very poor” as bad health and assigned a value of 2), and (7) the chronic disease information of respondents that can be obtained by asking: have you been diagnosed with hypertension/dyslipidemia/diabetes or high blood sugar/chronic lung diseases/liver disease/heart problems/stroke/kidney disease/stomach or other digestive diseases/arthritis or rheumatism/asthma by a doctor? If the respondents were informed by the doctor and know that he/she has one of these chronic diseases, the value is “1”; otherwise, it is “0”.

### Data analysis

Statistical analyses were performed using the SPSS Statistics 22.0 (IBM Corporation, Armonk, New York, USA). We performed a listwise deletion of the data with missing key variables. Some covariates contained missing values, and the proportion of missing values was < 5%. To ensure the completeness of the sample of key variables, we replaced the missing data with the mean of their integrity items (39). Frequency and case percentages were calculated to describe sociodemographic parameters and level distributions among participants.

Differences in characteristics between groups were investigated with chi-square tests for dichotomous variables. One-way between-group analyses of variance were employed to examine differences between depression subgroups and continuous variables (e.g., the number of chronic diseases). To fully appreciate the relationship between VI and depressive symptoms, the CES-D 10 was both dichotomized and continuous variable (CES-D10 cut-off ≥10) (34, 39). We assessed the association between VI and depression using multivariate logistic regression, adjusting for covariates and various mediators. Variance inflation factor (VIF) was used to measure multiple co-linearity in the logistic analysis; parameters with VIF≥10 were considered to be co-linear. Parameters with co-linearity were excluded from the logistic regression analysis. In the base model, adjustments were made for covariates including age, gender, area, education level, chronic disease, etc., Key outcomes were presented by odds ratios (ORs) and 95% confidence interval (95% CI). The chain-mediating effects of Internet use and social participation, as well as the moderating effect of Internet use frequency on the first half of the mediation pathway, were then tested using the PROCESS macro of SPSS 22.0 (model no. 6 and model no. 91), and a further test of the mediating effect was performed with the deviation proofreading method of bootstrap of non-parametric percentile (40). β-coefficient was used to describe the strength of association between paths, and statistical significance was set at *P* < 0.05 using a two-sided test.

## Results

### Sample characteristics

Among the 19,766 adult respondents in the fourth wave of the CHARLS, 17,433 respondents over the age of 45 years were selected. Of them, 42.0% [7,327] of the respondents had depressive symptoms and 32.1% (*n* = 5,589) reported that they had VI.

Table 1 presents the characteristics of participants by depression condition (non-depression group vs. depression group). Participants in the depression group were more female (46.6 vs. 60.5%), were older, and had worse self-reported health (15.8 vs. 39.2%), and only 57.9% had normal vision compared with those in the non-depression group. They were also found to be more likely to live in rural areas (77.0%) and have a relatively low level of education, with 71.9% having below middle school. In terms of Internet use, participants in the depression group were less likely to use the Internet (16.6 vs. 10.0%). Regarding social participation, depression participants were less exposed to different levels of social engagement, especially heavy group (18.9 vs. 14.6%).

**Table 1:** Basic characteristics of the sample.

**Variables**	**Total (*n* = 17,433)**	**Non-depression (*n* = 10,106)**	**Depression (*n* = 7,327)**	**Effect sizes^b^**	***P*-value**
**Gender, %**				0.138	0.000
Male	(8289) 47.5	(5398) 53.4	(2891) 39.5		
Female	(9144) 52.5	(4708) 46.6	(4436) 60.5		
**Age (years), %**				0.001	0.002
45–54	(5,006) 28.7	(3,013) 29.8	(1,993) 27.7		
55–64	(6,006) 34.5	(3,440) 34.0	(2,566) 35.0		
65–74	(4,680) 26.8	(2,662) 26.3	(2,018) 27.5		
≥75	(1,741) 10.0	(991) 9.8	(750) 10.2		
**Area, %**				0.010	0.000
Central of city/town	(3,468) 19.9	(2,312) 22.9	(1,156) 15.8		
Urban–rural integration zone	(1,470) 8.4	(940) 9.3	(530) 7.2		
Rural	(12,495) 71.7	(6,854) 67.8	(5,641) 77.0		
**Education level, %**				0.018	0.000
Below middle School	(11,258) 64.6	(5,992) 59.3	(5,266) 71.9		
Below college degree	(5,818) 33.4	(3,839) 38.0	(1,976) 27.0		
College degree and above	(360) 2.0	(275) 2.7	(85) 1.2		
**Marital status, %**				0.008	0.000
Married	(15,065) 86.4	(8,999) 89.0	(6,066) 82.8		
Divorced or widowed	(2219) 12.7	(1041) 10.3	(1178) 16.1		
Never married	(149) 0.9	(66) 0.7	(83) 1.1		
**Health status (self-reported), %**				0.264	0.000
Good	(12,962) 74.4	(8,506) 84.2	(4,456) 60.8		
Bad	(4,471) 25.6	(1,600) 15.8	(2,871) 39.2		
Chronic diseases (M±SD)	0.71 ± 1.03	0.60 ± 0.94	0.81 ± 1.11	0.204	0.000
**VI, %**				0.184	0.000
No	(11,844) 67.9	(7,605) 75.3	(4,239) 57.9		
Yes	(5,589) 32.1	(2,501) 24.7	(3,088) 42.1		
**Internet use, %**				0.094	0.000
No	(15,027) 86.2	(8,432) 83.4	(6,595) 90.0		
Yes	(2,406) 13.8	(1,674) 16.6	(732) 10.0		
**Social participation, %**				0.004	0.000
No	(8,697) 49.9	(4,807) 47.6	(3,890) 53.1		
Mild	(3,322) 19.1	(1,943) 19.2	(1,379) 18.8		
Moderate	(2,434) 14	(1,446) 14.3	(988) 13.5		
Heavy	(2,980) 17	(1,910) 18.9	(1,070) 14.6		

Note: ^a^VI: vision impairment. ^b^Effect sizes: chi-square test using Cramer's, ANOVA using η^2^, and *t*-test adapted to Cohen's d.

### The relationship between VI and depression

Table 2 reveals that after controlling for sociodemographic characteristics, health status, and chronic diseases, as expected, people who got VI had significantly higher odds of depression (OR = 1.61, 95% CI: 1.50–1.73). In terms of other significant covariates, female was associated with higher odds of depression (OR = 1.55, 95% CI: 1.45–1.65). When compared with respondents living in the central city/town, those living in rural areas had greater odds of depression (OR = 1.38, 95% CI: 1.26–1.50). Moreover, being divorced or widowed, being never married, having bad health status, and having more chronic diseases were associated with higher odds of depression. Being in 65–74 years and ≥75 years and having below college degree and college degree and above were associated with lower odds of depression.

**Table 2:** Association of VI and depression: Logistic regression results.

**Variable**	**Depression, OR (95% CI)**	***P* value**
**VI**		0.000
No	1 (ref)	
Yes	1.61(1.50–1.73)	
**Gender**		0.000
Male	1 (ref)	
Female	1.55 (1.45–1.65)	0.000
**Age (years)**		
45-54	1 (ref)	
55–64	1.03 (0.95–1.12)	0.505
65–74	0.85 (0.78–0.93)	0.000
≥75	0.76 (0.67–0.87)	0.000
**Area**		
Central of city/town	1 (ref)	
Urban-Rural Integration Zone	1.06 (0.92–1.21)	0.413
Rural	1.38 (1.26–1.50)	0.000
**Education level**		
Below middle School	1 (ref)	
Below college degree	0.79 (0.73–0.85)	0.000
College degree and above	0.63 (0.49–0.82)	0.001
**Marital status**		
Married	1 (ref)	
Divorced or widowed	1.47 (1.33–1.63)	0.000
Never married	1.84 (1.30–2.60)	0.001
**Health status (self-reported), %**		
Good	1 (ref)	
Bad	2.63 (2.44–2.84)	0.000
Chronic diseases	1.15 (1.11–1.19)	0.000

Note: VI: vision impairment.

### Verification of chain-mediating effect

The results showed that there are significant relationships between VI, Internet use, social participation, and depression. After controlling for gender, age, region, educational level, marital status, health status, and chronic diseases, the analysis chain-mediating effects of Internet use and social participation in the relationship between VI and depression. The results confirmed that Internet use and social participation played a partial mediating role between VI and depression, i.e., when VI influenced depression, part of it was direct and part went through the mediating variables Internet use and social participation (Supplementary Table 1).

**Figure 1:**
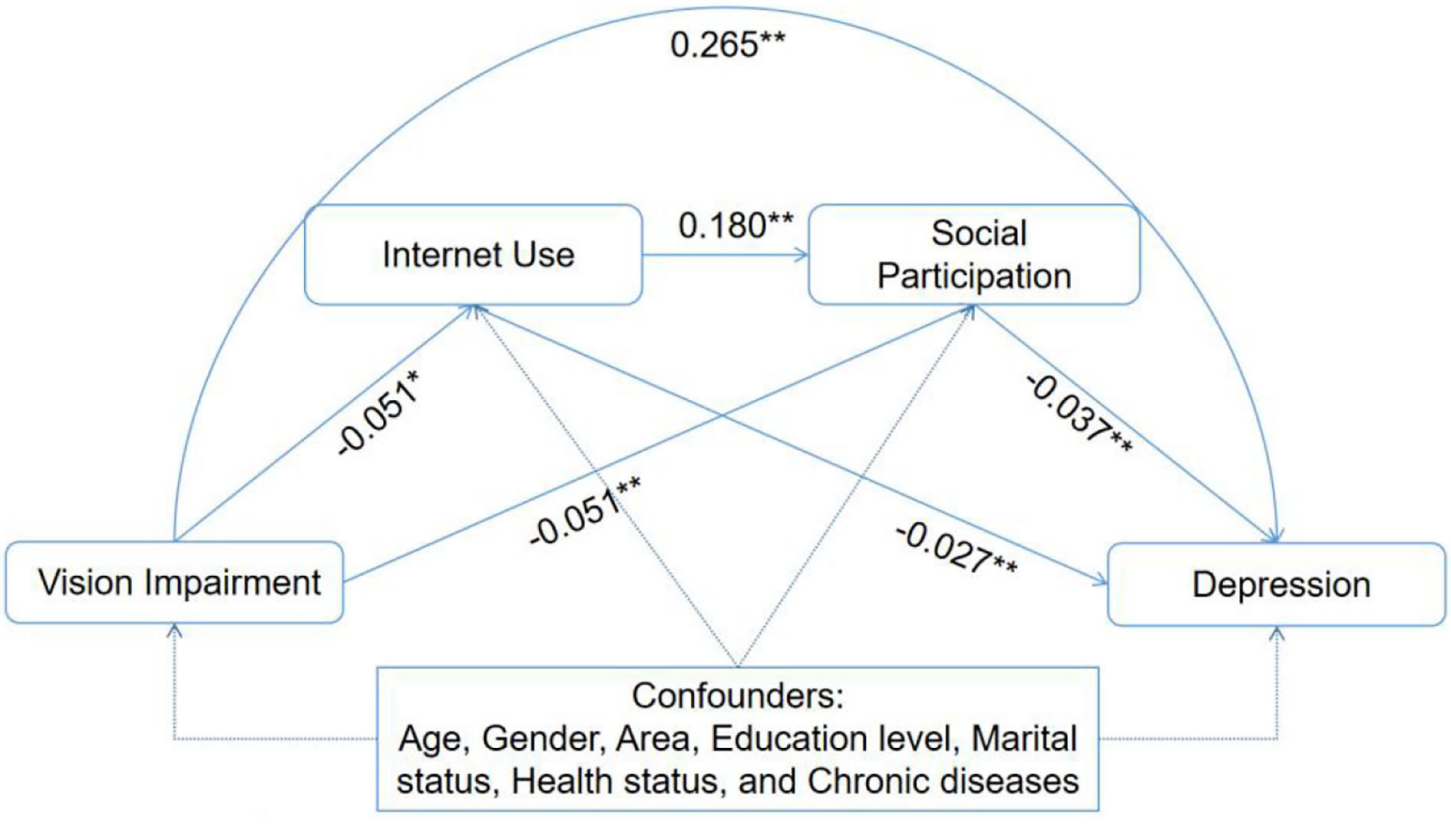
Statistical model of chain-mediating effect.

The present study then further examined the path and proportion of the mediating effect by using the bias-corrected non-parametric percentile bootstrap method (Table 3). With VI as the independent variable, depression as the dependent variable, and Internet use and social participation as the mediating variables, the bootstrap sampling number was 5,000, and a 95% confidence interval was set. The results showed that VI effectively predicted depression (positive), Internet use (negative) and social participation (negative), Internet use effectively predicted social participation (positive) and depression (negative), and social participation effectively predicted depression (negative) (Figure 1).

**Table 3:** The proportion of mediating effect.

	**Effect**	**Boot SE**	**Boot LLCI**	**Boot ULCI**	**% of the total effect (%)**	**% of total mediating effect (%)**
Total mediating effect	0.017	0.004	0.009	0.025	1.33	100.00
Ind1:VI->Internet use->Depression	0.006	0.002	0.002	0.012	0.50	37.72
Ind2:VI->Social participation->Depression	0.009	0.003	0.003	0.015	0.70	52.69
Ind3:VI->Internet use->Social participation->Depression	0.002	0.001	0.001	0.003	0.13	9.58

Note: Boot SE is the standard error for estimating indirect effects by the percentile bootstrap method of bias correction. Boot CI lower limit and boot CI upper limit refer to the lower limit and upper limit of 95% confidence interval, respectively.

The results of the test for mediating effects are given in Table 3. VI had significant direct effects on the prediction of depression (effect = 0.017, 95% CI: 0.009, 0.025), Internet use (effect = 0.006, 95% CI: 0.002, 0.012), and social participation (effect = 0.009, 95% CI: 0.003, 0. 015) and a significant chain-mediating effect of both (effect = 0.002, 95% CI: 0.001, 0.003), with the total indirect (mediating) effect accounting for 1.33% of the total effect; the proportions of Internet use, social participation, and chain-mediating effects in the total mediating effect were 37.72, 52.69, and 9.58%, respectively.

### Verification of moderated mediating effect

To determine whether the chain-mediating effects of Internet use and social participation in the relationship between VI and depression differ with the frequency of Internet use, we validated the moderated mediation model using PROCESS Macro model no. 91. It was confirmed that the indirect effects of VI on depression through Internet use and social participation did not vary with the frequency of Internet use (Supplementary Table 2 and Figure 1).

The overall size of the conditional indirect effects of this study, that is, the index of moderated mediation, was 0, and the 95% bootstrap CI for this was [−0.001, 0.0008]. As the 95% CI was 0, the moderated mediating effect was not significant.

## Discussion

The present study used a novel approach to examine the interaction effects of Internet use and social participation for depression in patients with VI, helping shed light on the targeted feasible responses for the vulnerable population. Some previous studies have identified a cross section between VI and depression (41, 42) and longitudinal (43) relationship. The formation pathway in the relationship between VI and depression has not been systematically studied. Especially with the development of social and network technology, the effect of the Internet and social participation on depression is more worthy of attention. Therefore, the present findings provided a more comprehensive pathway to the relationship between VI and depression and analyzed the mediating role of Internet use and social participation in the relationship between VI and depression.

This study investigated 17,433 Chinese participants using cross-sectional data from a nationally representative cohort of middle-aged and older adults in China. The findings suggested an association between VI and depression, controlling for gender, age, region, education level, marital status, health level, and chronic disease. This association was attenuated and mitigated when mediating factors were added to the regression, indicating a significant negative mediating effect of mediating factors. A certain percentage (2.74%) of the effect of VI on depression was mediated by two sets of explanatory factors. In the chain-mediated analysis, social participation had the largest effect on the VI–depression association, with an indirect effect of 1.85%, followed by Internet use (0.63%) and a chain mix of both factors (0.27%). Furthermore, this study examined the moderating mediating effect of Internet use frequency and found it to be not statistically significant.

Our findings have shown that VI is strongly associated with depression, which is consistent with other research in different regions. Several reports showed a relatively consistent association between VI and mental health among different age populations. Cosh (44) surveyed the Norwegian population over 60 years and found that vision loss would lead to an increase in depressive symptoms over time and bring additional long-term risks to depression severity. In addition, Xiaowei Dong et al. reported that the participants with VI had 43% higher odds of depression than those with normal vision in China (10). Although the reasons for this result were not fully understood, the activity limitation model of depressive emotions posited that chronic health conditions could lead to depression, partly as individuals' social participation and daily activities are restricted (42, 45). The social participation of middle-aged and older adults with VI was related to the individual's physical and mental health (41), which highlighted the necessity of lifestyle modification, especially among those with severe VI who were invulnerable to social participation and daily activities (41, 45).

Our results also provided support for the hypothesized mediation model in which increased levels of Internet use and social participation lead to lower levels of depression, which we found to be partially mediated—populations with VI who are accompanied by some level of Internet use and social participation have a correspondingly mitigated risk of depression. Dovetailing with the research about how both Internet and social factors are important significant control factors of depression (46, 47), our results suggested that the Internet and social engagement play a key role in the mental health of older adults. This is consistent with previous research, which indicated that Internet use leads to improvements in social connectedness, social support, mental health, and depression in older adults (48, 49).

Aging highlights the revelation that more older adults suffer from depression and social isolation, and both online and offline social engagement activities provide us with effective alternatives. On the one hand, our findings suggested that measures to encourage appropriate Internet use among older adults, especially those with VI, may prevent depression in this population. Contrary to previously published studies, Banjanin et al. believed that Internet use was positively correlated with depressive symptoms in adolescents (47). The reason for this result may be that the target population and age of the two studies were not consistent, and there were differences in Internet usage habits and time. Correspondingly, several studies have focused on the role of the Internet in relieving the symptoms of depression in older adults. Atsushi et al. (48) pointed out that online communication with family and friends had a significant role in preventing clinical depression in older adults, especially during the new coronary pneumonia crisis. Chatting and watching videos online could significantly alleviate the loneliness of middle-aged and older adults and improve their social participation ability, and the use of the Internet for communication purposes was associated with better mental health (21). For the VI group, the intelligent call, barrier-free voice conversion function, and screen magnification function provided by the Internet could narrow the gap between them and the normal population and strengthen its sense of social participation. Notably, Internet-based eHealthcare and mHealth could provide patients with VI with timely and convenient vision testing, diagnosis, and treatment services, which could effectively alleviate their anxiety and depression caused by the disease (50).

On the other hand, we found that a range of offline social participation may also be a key variable in mitigating the negative association between VI and depression. The study showed that older adults involved in social activities, volunteer work, and donations had a reduced risk of depressive symptoms, while more frequent and diverse participation activities further reduced the risk (51). Furthermore, the current findings are consistent with previous research by Yanni et al. (52), who showed in a previous study that the combination of full-time work and volunteer activities was particularly protective against depression compared with any activity alone. Related to this, we found an additional beneficial effect of participating in both online and offline social activities. The more types of activities older adults participated in, the less likely they were to experience depressive symptoms. This finding is consistent with role accumulation theory, which suggests that occupying multiple roles helps individuals experience more social networks, resources, and self-esteem (53). Not only the frequency of engagement but also the diversity of activity types is important (54).

Furthermore, while Internet use and social participation may alleviate depression in VI, it is important to note that only 50.1% of the Chinese middle-aged and older adults reported engaging in offline social engagement and only 13.8% had Internet use habits. These results appear to be consistent with several other studies focusing on different populations (55, 56). Thus, this suggests the need for further attention to a range of social engagement activities between middle-aged and older adults, especially the VI group. In terms of offline activities, the current Chinese government has proposed a series of measures to encourage middle-aged and older adults to actively engage in social participation. For example, it is exploring flexible employment models for older adults, encouraging localities to establish a talent database for older adults, providing career guidance services for older adults who are willing to work, carrying out in-depth “Silver Age Action,” and guiding the older adults to actively participate in community activities in the form of volunteer services. Concerning further trends in the use of online Internet features, policymakers should consider supporting research and development of assistive technologies and designs to help middle-aged and older Internet users overcome health-related difficulties in using the Internet, such as redesigning interfaces to use larger fonts and simpler layouts (57), but more work is needed to understand the needs of these middle-aged and older adults. For the VI group, it is almost certain that they are limited by their health status and rarely use the Internet. Therefore, they certainly need to understand the current applications and value of the Internet in alleviating depression and providing medical care. Perhaps, convenient teleconsultation on the Internet could provide them with the medical and health services they need.

The study has a few limitations. First, VI was self-reported, with no data on the timing of onset or further disease progression, while depression was based on CES-D10 judgment rather than a medical diagnosis. Therefore, we do not know the trends in VI and depression over time. Second, only cross-sectional data on Internet use were collected in this study, and the cross-sectional study design does not allow for the establishment of causal relationships and restricts chronological order, which means that the findings need to be interpreted with caution. Third, there are still untested mediators.

## Conclusions

Both online and offline social participation has shown a mitigating effect on improving depression in middle-aged and older adults, especially in the population with VI. As more and more daily social activities (e.g., living payments, online shopping, financial management, etc.,) and medical care services require the use of the Internet, it is becoming increasingly important to promote Web-based technology for social engagement behaviors and medical care behaviors between middle-aged and older adults. Based on a large nationwide cohort in China, Internet use and social engagement were found to play a mediating utility in the effect of VI on depression. Therefore, targeted Internet services and social engagement promotion services should be designated for middle-aged and older adults, especially those with VI, and the inequities caused by other social determinants for those with VI should be considered.

## Data availability statement

The original contributions presented in the study are included in the article/Supplementary material, further inquiries can be directed to the corresponding author.

## Ethics statement

The China Health and Retirement Longitudinal Study (CHARLS) was a survey approved by the Ethical Review Committee of Beijing University, and all participants signed informed consent at the time of participation. There is no need for additional ethics approval for the approved data users.

## Author contributions

WH and WZ conceptualized the study. WH, PL, YG, and XQ collected and analyzed the data. WH and PL wrote the manuscript. WZ, JC, and JY revised and finalized the manuscript. All authors contributed to the article and approved the submitted version.

## Funding

This study was supported by the National Science Foundation of China (Grant No. 81871061), 1·3·5 Project for Disciplines of Excellence, West China Hospital, Sichuan University (No. ZYJC21004), and Clinical Research Innovation Project from West China Hospital, Sichuan University (Grant No. 2019HXCX03).

## Conflict of interest

The authors declare that the research was conducted in the absence of any commercial or financial relationships that could be construed as a potential conflict of interest.

## Publisher's note

All claims expressed in this article are solely those of the authors and do not necessarily represent those of their affiliated organizations, or those of the publisher, the editors and the reviewers. Any product that may be evaluated in this article, or claim that may be made by its manufacturer, is not guaranteed or endorsed by the publisher.
