# Supplementary material for: Self-reported visual impairment and depression of middle-aged and older adults: The chain-mediating effects of internet use and social participation
Source: Front Public Health. 2022 Nov 17;10:957586. doi: 10.3389/fpubh.2022.957586 (PMC9714326; doi:10.3389/fpubh.2022.957586)
Supplement: Supplementary file 1 [file Data_Sheet_1.DOCX]

Supplementary Material

# Supplementary Table 1

Chain mediating effects of Internet function use and social participation in the relationship between VI and depression

| **Regression equation** | | **Overall fit index** | | | **Regression Coefficient Significance** | | |
| --- | --- | --- | --- | --- | --- | --- | --- |
| **Outcomes** | **Predictors** | **R** | **R**2 | **F** | **B** | **t** | **P** |
| **Depression** | VI | 0.405 | 0.164 | 342.597 | 0.265 | 17.123 | 0.000 |
|  | Internet function use |  |  |  | -0.027 | -2.875 | 0.004 |
|  | Social participation |  |  |  | -0.037 | -8.848 | 0.000 |
|  | Gender |  |  |  | 0.122 | 17.083 | 0.000 |
|  | Age |  |  |  | -0.053 | -7.132 | 0.000 |
|  | Area |  |  |  | 0.054 | 6.989 | 0.000 |
|  | Education level |  |  |  | -0.057 | -6.940 | 0.000 |
|  | Marital status |  |  |  | 0.070 | 9.747 | 0.000 |
|  | Health status |  |  |  | 0.240 | 31.920 | 0.000 |
|  | Chronic Diseases |  |  |  | 0.078 | 10.940 | 0.000 |
| **Internet function use** | VI | 0.419 | 0.176 | 463.865 | -0.051 | -3.289 | 0.001 |
|  | Gender |  |  |  | -0.006 | -0.812 | 0.417 |
|  | Age |  |  |  | -0.176 | -24.103 | 0.000 |
|  | Area |  |  |  | -0.159 | -21.606 | 0.000 |
|  | Education level |  |  |  | 0.251 | 32.666 | 0.000 |
|  | Marital status |  |  |  | 0.007 | 0.909 | 0.363 |
|  | Health status |  |  |  | -0.048 | -6.485 | 0.000 |
|  | Chronic Diseases |  |  |  | 0.022 | 3.023 | 0.003 |
| **Social participation** | VI | 0.261 | 0.068 | 141.288 | -0.051 | -0.504 | 0.614 |
|  | Internet function use |  |  |  | 0.180 | 21.747 | 0.000 |
|  | Gender |  |  |  | 0.044 | 4.807 | 0.000 |
|  | Age |  |  |  | -0.014 | -1.787 | 0.074 |
|  | Area |  |  |  | -0.056 | -7.349 | 0.000 |
|  | Education level |  |  |  | 0.092 | 10.240 | 0.000 |
|  | Marital status |  |  |  | 0.005 | 0.487 | 0.626 |
|  | Health status |  |  |  | -0.043 | -6.208 | 0.000 |
|  | Chronic Diseases |  |  |  | 0.027 | 3.025 | 0.003 |

VI: vision impairment

# Supplementary Table 2

Moderating role of Internet usage frequency in chain mediation

| Regression equation | | Overall fit index | | | Regression Coefficient Significance | | |
| --- | --- | --- | --- | --- | --- | --- | --- |
| Outcomes | Predictors | R | R^2^ | F | B | t | P |
| **Depression** | VI | 0.405 | 0.164 | 342.600 | 1.236 | 17.006 | 0.000 |
|  | Internet function use |  |  |  | -0.107 | -3.438 | 0.001 |
|  | Social participation |  |  |  | -0.025 | -5.163 | 0.000 |
|  | Gender |  |  |  | 1.136 | 16.957 | 0.000 |
|  | Age |  |  |  | -0.257 | -7.069 | 0.000 |
|  | Area |  |  |  | 0.313 | 7.195 | 0.000 |
|  | Education level |  |  |  | -0.510 | -7.185 | 0.000 |
|  | Marital status |  |  |  | 0.871 | 9.723 | 0.000 |
|  | Health status |  |  |  | 2.564 | 32.083 | 0.000 |
|  | Chronic Diseases |  |  |  | 0.352 | 10.860 | 0.000 |
| **Internet function use** | VI | 0.419 | 0.176 | 463.865 | -0.059 | -3.289 | 0.001 |
|  | Gender |  |  |  | -0.014 | -0.812 | 0.417 |
|  | Age |  |  |  | -0.213 | -24.103 | 0.000 |
|  | Area |  |  |  | -0.230 | -21.606 | 0.000 |
|  | Education level |  |  |  | 0.556 | 32.666 | 0.000 |
|  | Marital status |  |  |  | 0.020 | 0.909 | 0.363 |
|  | Health status |  |  |  | -0.128 | -6.485 | 0.000 |
|  | Chronic Diseases |  |  |  | 0.024 | 3.023 | 0.003 |
| **Social participation** | VI | 0.270 | 0.073 | 124.459 | -0.351 | -3.095 | 0.002 |
|  | Internet function use |  |  |  | 1.049 | 11.484 | 0.000 |
|  | Internet usage frequency |  |  |  | -0.024 | -0.107 | 0.915 |
|  | Internet function use*Internet usage frequency |  |  |  | 0.034 | 0.285 | 0.776 |
|  | Gender |  |  |  | 0.606 | 5.789 | 0.000 |
|  | Age |  |  |  | -0.098 | -1.728 | 0.084 |
|  | Area |  |  |  | -0.481 | -7.073 | 0.000 |
|  | Education level |  |  |  | 1.207 | 10.911 | 0.000 |
|  | Marital status |  |  |  | 0.083 | 0.593 | 0.554 |
|  | Health status |  |  |  | -0.870 | -6.902 | 0.000 |
|  | Chronic Diseases |  |  |  | 0.148 | 2.847 | 0.004 |

VI: vision impairment.

# Supplementary Figure 1


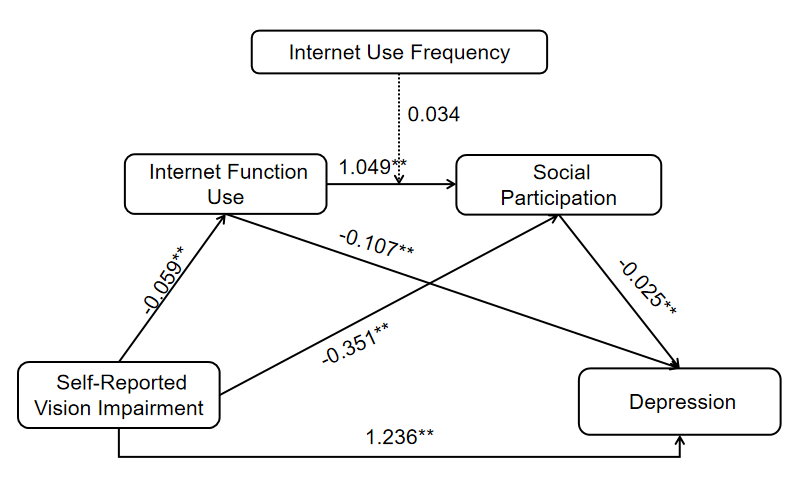


**Supplementary Figure 1. Statistical model of moderated mediation. ** P < 0.01**

# 
